# Teriparatide relieves ovariectomy-induced hyperalgesia in rats, suggesting the involvement of functional regulation in primary sensory neurons by PTH-mediated signaling

**DOI:** 10.1038/s41598-020-62045-4

**Published:** 2020-03-24

**Authors:** Tomoya Tanaka, Ryoko Takao-Kawabata, Aya Takakura, Yukari Shimazu, Momoko Nakatsugawa, Akitoshi Ito, Ji-Won Lee, Koh Kawasaki, Tadahiro Iimura

**Affiliations:** 1 Pharmaceuticals Research Center, Asahi Kasei Pharma Corporation, 632-1 Mifuku, Izunokuni city, Shizuoka, 410-2321 Japan; 2 Department of Pharmacology, Graduate School of Dental Medicine, Hokkaido University, N13 W7, Sapporo, 060-8586 Japan; 3 Division of Bio-Imaging, Proteo-Science Center (PROS), Ehime University, Shitsukawa, Toon city, Ehime 791-0295 Japan

**Keywords:** Hormone receptors, Osteoporosis

## Abstract

Clinical studies have reported that teriparatide (TPTD), a human parathyroid hormone analog, reduces back pain in osteoporotic patients. However, the mechanistic insights of this pharmacological action remain elusive. This study investigated the antinociceptive effect of TPTD mainly on primary sensory neurons in ovariectomized (OVX) rats. The plantar test showed thermal hyperalgesia in the OVX rats, which was significantly, but not fully, recovered immediately after the initial TPTD administration. The von Frey test also demonstrated reduced withdrawal threshold in the OVX rats. This was partially recovered by TPTD. Consistently, the number and size of spinal microglial cells were significantly increased in the OVX rats, while TPTD treatment significantly reduced the number but not size of these cells. RNA sequencing-based bioinformatics of the dorsal root ganglia (DRG) demonstrated that changes in neuro-protective and inflammatory genes were involved in the pharmacological effect of TPTD. Most neurons in the DRG expressed substantial levels of parathyroid hormone 1 receptor. TPTD treatment of the cultured DRG-derived neuronal cells reduced the cAMP level and augmented the intracellular calcium level as the concentration increased. These findings suggest that TPTD targets neuronal cells as well as bone cells to exert its pharmacological action.

## Introduction

More than 80% of osteoporotic patients reportedly have low back pain, which leads to disability and progression of bone and muscle weakening, and eventually reduces quality of life^1,2^. This osteoporotic pain occurs in patients regardless of obvious bone fractures in the vertebrae and other sites. A possible causative mechanism of osteoporotic pain is chronic neuronal excitement in intraosseous sensory nerve systems by acids and inflammatory cytokines produced by the activation of osteoclasts, as well as monocytes and macrophages, due to estrogen deficiency^3,4^. This mechanism has been proposed to be involved in cancer-associated bone pain patients with bone metastases^5^. Osteoporotic patients may also experience pain originating from collapsed vertebral bodies, degenerated intervertebral disc and facet joints^6^. Chronification of osteoporotic pain involves hypoactivity of the descending inhibitory nerve system that modulates ascending pain-transmission in the spinal cord with the decreased expression of serotonin receptors^3,7^.

Anti-osteoporotic agents have been reported to be clinically and experimentally effective against bone pain. Bisphosphonates (BPs) exerted pain-modulating effects by suppressing osteoclasts, monocytes, and macrophage activity to curb the production of acids and inflammatory cytokines^3,8^. It has also been reported that the BP drug minodronate exerts a pain-modulating effect by inhibiting the purinergic P2X2/3 receptor^9^. Calcitonin depresses bone resorption and osteoclast activity by acting on osteoclast surface receptors. It has also been reported that calcitonin exerts its pain-modulating effect by depressing osteoclasts and correcting the decline in serotonin receptor activity that causes hypoactivity of the descending inhibitory system^7,10,11^. To study the antinociceptive effect of anti-osteoporotic agents, ovariectomized (OVX) animals, a widely-used osteoporosis animal model, are often used. It is well established that OVX animals show not only loss of bone density but also hyperalgesia to heat and mechanical stimuli^8,10–13^. It has been reported that estradiol improves the pain-related behavior induced by OVX, indicating that estrogen deficiency influences the response to heat and mechanical stimuli^11–13^. Also, It has been reported that the pain-related behavior induced by OVX is improved not only by BP and calcitonin but also by pregabalin and morphine^8,10,11,14^.

Teriparatide (TPTD; the 1–34 fragment of human parathyroid hormone [PTH]) exerts a bone anabolic effect and is a clinically effective therapeutic agent for osteoporosis by acting on PTH 1 receptor (PTH1R) in osteoblast-lineage cells, promoting the proliferation, differentiation, and survival of bone-forming osteoblasts. Accumulated epidemiological studies have reported that TPTD treatment suppresses the onset and modulates the exacerbation of back pain in osteoporotic patients^15–20^. TPTD therapy was also effective against post-vertebral fracture pain^21–23^ and incurable back pain in patients treated with BPs^24^. Improvement of back pain by TPTD appeared to be observed in earlier stages of the treatment than with calcitonin treatment^25^. However, despite the existence of multiple clinical reports as mentioned above, the pharmacological mechanism of the anti-osteoporotic pain effect of TPTD remains unclear.

This study investigated the antinociceptive effect of TPTD in OVX rats. We conducted pain-related behavioral tests and transcriptome analyses in the dorsal root ganglia (DRG) collected from the model rats followed by bioinformatic analyses.

## Results

### Effects of TPTD on the pain-related behavior in OVX rats

**Figure 1:**
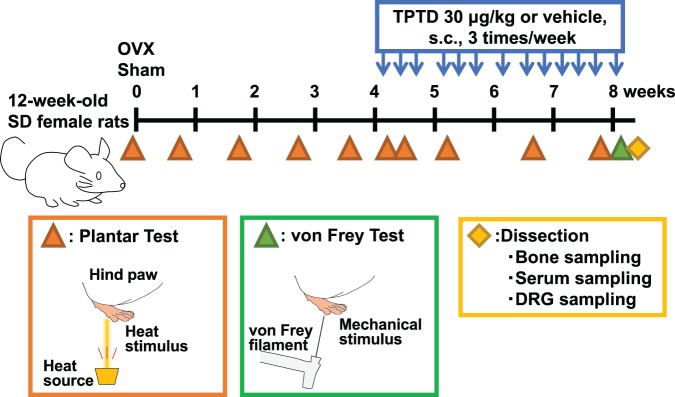
The time schedule of the experiments and sampling. The time schedule of OVX, TPTD administration, behavioral tests (plantar test and von Frey test), and sampling are shown.

**Figure 2:**
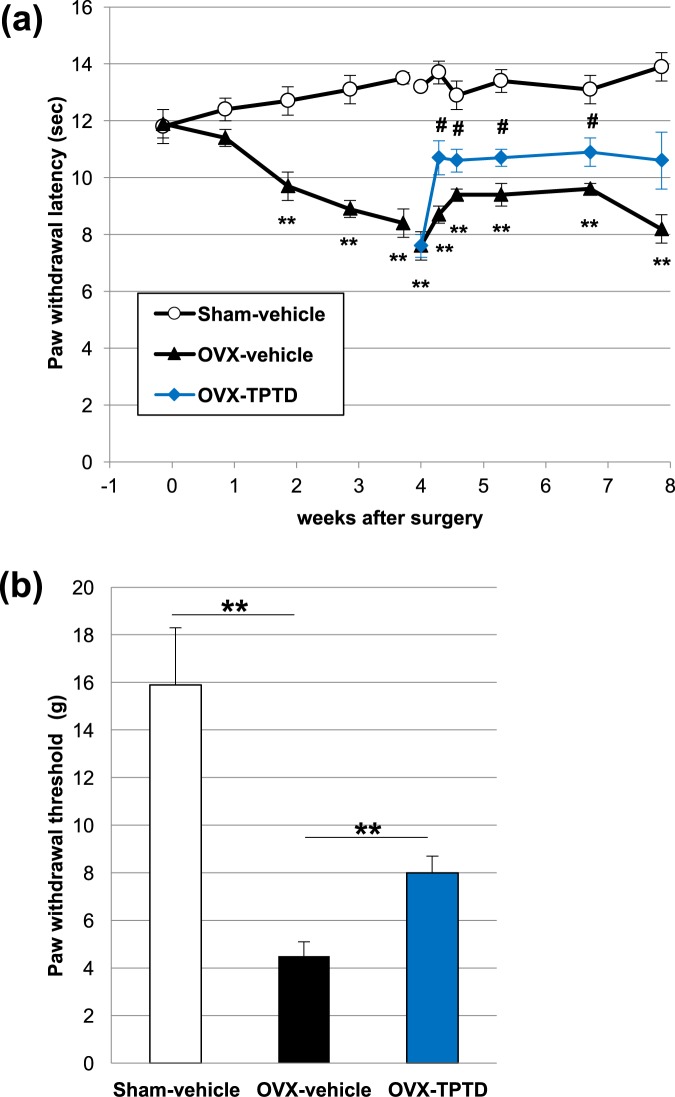
Antinociceptive effect of TPTD in OVX rats. (**a**) The plantar test was conducted during the period of the TPTD administration. TPTD administration (in the OVX-TPTD group) promptly and significantly recovered the reduced latency (shown in blue line). Significant difference: **p < 0.01 vs. sham, #p < 0.05 vs. OVX-vehicle group (a two-way ANOVA with a post-hoc Student’s *t*-test). (**b**) The von Frey test was carried out 4 weeks after the TPTD administration. The withdrawal threshold in the OVX-TPTD group (filled column in blue) was significantly higher than that in the OVX-vehicle group (filled column) at 4 weeks after the initial TPTD administration. Significant difference: **p < 0.01. Data are presented as the means ± S.E.M.

We first conducted 2 pain-related behavioral tests (the plantar test and von Frey test) in the 3 experimental groups of Sham-vehicle, OVX-vehicle, and OVX-TPTD (Figs. 1 and 2). The paw withdrawal latency in the OVX-vehicle group gradually shortened and was significantly shorter than that in the Sham-vehicle group at 2 weeks after surgery on the plantar test (p < 0.01, *t*-test) (Fig. 2a). Significant differences in the latency between these 2 groups were continuously observed up to 8 weeks after the surgery, indicating that OVX induced prolonged hyperalgesia. At 4 weeks postoperatively, the OVX-TPTD group underwent TPTD administration (30 μg/kg) at 3 times a week for 4 weeks. At 6 h after the initial TPTD administration, the latency of the OVX-TPTD group immediately recovered to 40% of that in the Sham-vehicle group, a significant difference compared with that in the OVX-vehicle group (p < 0.05, *t*-test), until the end of the analysis (8 weeks after the surgery). The latency of the OVX-TPTD group showed an increasing trend at 4 weeks after the initial administration (p = 0.051, *t*-test), and the antinociceptive effect remained about the same through the final day of administration.

Consistently, the von Frey test at 4 weeks after the initial administration (8 weeks postoperatively) showed that the OVX-vehicle group had a significantly lower withdrawal threshold to mechanical stimuli than the Sham-vehicle group (p < 0.01, *t*-test). The withdrawal threshold of the OVX-TPTD group was significantly higher than that of the OVX-vehicle group (p < 0.01, *t*-test), which indicated that TPTD recovered the lowered threshold by OVX to approximately 30% of that in the Sham-vehicle group (Fig. 2b).

### Bone anabolic effects of TPTD treatment in the OVX rats

**Figure 3:**
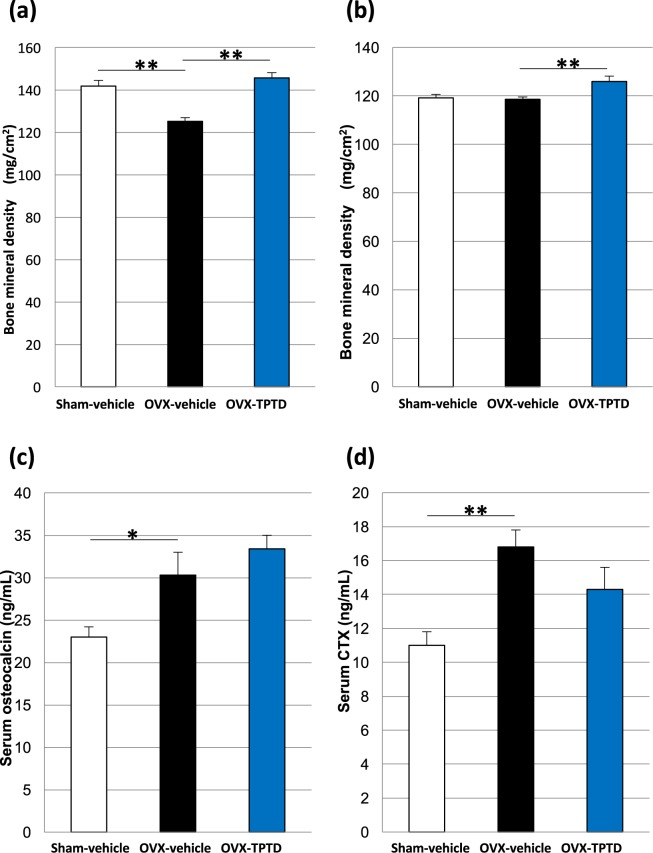
Anabolic effects of TPTD on bone metabolism in OVX rats. The BMD of the distal femur (**a**) and the femoral shaft (**b**) were measured at 4 weeks after the initial TPTD or vehicle administration. TPTD treatment significantly recovered the BMD in the OVX-TPTD group (filled column in blue). The serum osteocalcin concentration (**c**) and serum CTX concentration (**d**) were measured 4 weeks after the initial administration. Significant difference: *p < 0.05, **p < 0.01. Data are presented as the means + S.E.M.

We next confirmed the pharmacological effects of TPTD treatment on bones in the OVX rats (Fig. 3) at 4 weeks after the initial TPTD treatment. The distal femoral bone mineral density (BMD) in the OVX-TPTD group was significantly greater than that in the OVX-vehicle group and comparable to those in the Sham-vehicle group (Fig. 3a). The BMD of the femoral shaft in the OVX-TPTD group was higher than that in the OVX-vehicle group and Sham-vehicle group (Fig. 3b). Therefore, the bone anabolic effects of TPTD treatment in this experimental regimen was confirmed.

The serum levels of osteocalcin and CTX were significantly augmented in the OVX-vehicle group compared with the Sham-vehicle group, suggesting that bone turnover was stimulated by OVX (Fig. 3c,d). TPTD treatment up- and downregulated these bone formation and resorption makers, respectively, compared to those in the OVX-vehicle group, albeit not significantly.

### Effects of TPTD on microglial cells reside in spinal cord in the OVX rats

**Figure 4:**
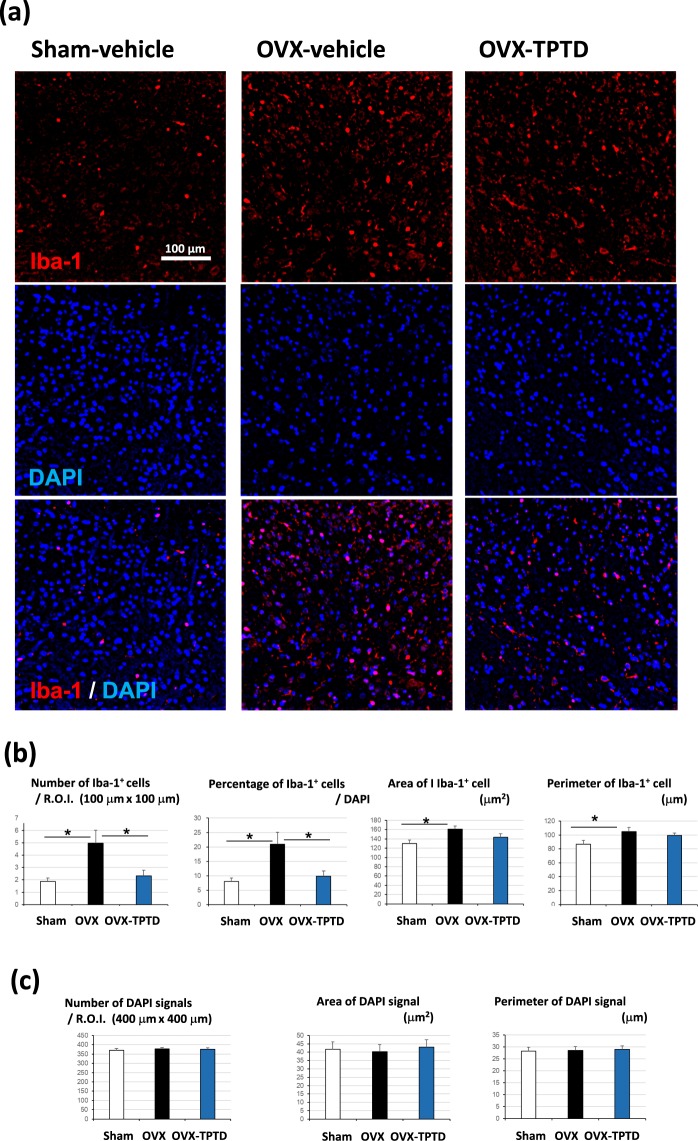
The effect of TPTD on spinal microglia. (**a**) Iba-1-positive microglial cells were detected by fluorescence immunohistochemistry. Representative specimens from three experimental groups that show the scores closest to the mean value of the number of Iba-1-positive cells (shown in b) are shown. (**b, c**) The number and morphometrical parameters such as area and perimeter of Iba-1 and DAPI signals were scored and compared. Significant difference: *p < 0.05. Data are presented as the means + S.E.M.

To further confirm the antinociceptive effect of TPTD in the OVX rats. We carried out immunohistochemical staining using anti-Iba-1 antibodies on tissue sections of spinal cord obtained from specimens of 3 experimental groups, and compare spatial distribution pattern of Iba-1-positive microglial cells (Fig. 4, Supplementary Figure 1a), since it is well established that peripheral nerve injury-induced hyperalgesia involves proliferation and morphological hypertrophy of microglia in the central nervous system^26,27^. The number of Iba-1-positive microglial cells in dorsal horn area (per area, and percentage in total cells) were significantly increased in the OVX-vehicle group compared to that in the Sham-vehicle group (p < 0.05, *t*-test) (Fig. 4b). This number in the OVX-TPTD group was significantly reduced (p < 0.05, *t*-test), but not reached to the level in the Sham-vehicle group. Morphometrical parameters such as area and perimeter of microglial cells were significantly higher in the OVX-vehicle group than those in the Sham-vehicle group (p < 0.05, *t*-test). TPTD treatment did not significantly affected on these morphometrical parameters. The number of DAPI-positive cellular nuclei and their morphometrical parameters did not show significant differences among 3 experimental groups (Fig. 4c). Specificity of immunohistochemical staining using anti-Iba-1 antibodies was confirmed by conducting simultaneous negative control experiments using nonspecific IgGs (Supplementary Figure 1b).

### Existence of PTH1R in rat DRG neurons

**Figure 5:**
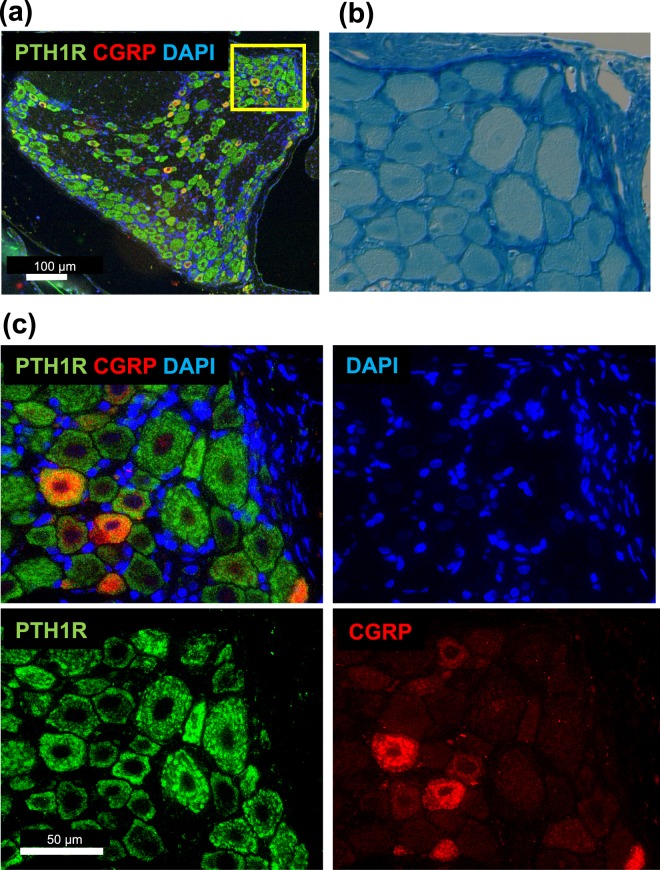
Detection of PTH1R in DRG neurons. (**a**) PTH1R was detected by fluorescence immunohistochemistry in DRGs, including CGRP-positive and CGRP-negative neurons. (**b**) Klüver-Barrera staining of the rectangle-bordered area shown in (**a**) for a morphological comparison. (**c**) Fluorescence immunohistochemistry of the rectangle-bordered area. Fluorescence signals of DAPI, PTH1R and CGRP are shown in blue, green and red, respectively.

To investigate whether or not DRG contains responsive cells to TPTD, we analyzed PTH1R by immunohistochemistry (Fig. 5). We also used anti- calcitonin gene-related protein (CGRP) as a marker of peptidergic neurons and anti-NF200 as a marker of myelinated neurons to dissociate neuronal subtypes in rat DRGs. PTH1R was detected in most neurons of the DRG, regardless of neuronal type. The transcription of *Pth1r* was detected by polymerase chain reaction (PCR) in isolated rat DRG (Supplementary Figure 2). PTH 2 receptor (PTH2R) was also detected by immunohistochemistry in most DRG neurons but not in bone (Supplementary Figure 3). Immunohistochemistry using anti-PTH1R showed signals in the cells covering the bone surface in vertebral bone tissue, thus confirming its expression in osteoblast-lineage cells (Supplementary Figure 4). Specific immunostaining with antibodies against PTH1R and CGRP was confirmed by conducting simultaneous negative control experiments using nonspecific IgGs (Supplementary Figure 5).

### Changes in the DRG transcriptome profiles by OVX and TPTD treatment

**Figure 6:**
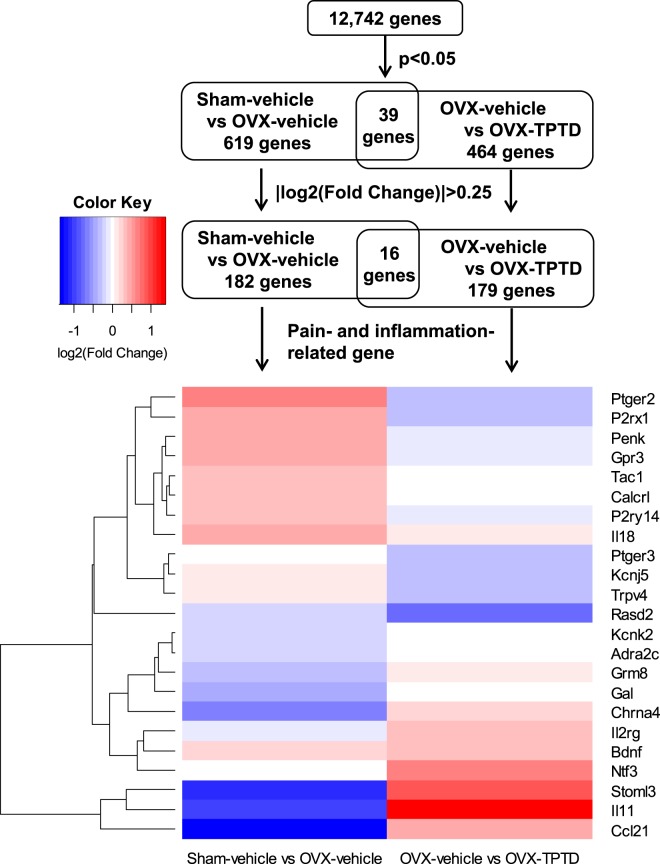
An analytical flowchart of gene-expression analyses by RNA-seq. RNA samples isolated from DRGs collected from the experimental groups were subjected to RNA-seq. A total of 12,742 genes were initially analyzed. Between the Sham-vehicle group and OVX-vehicle group, 182 genes were identified with statistical significance and fold change, while 179 genes were identified between the OVX-vehicle group and OVX-TPTD group. Pain- and inflammation-related genes were selected from the list of the differentially expressed genes. The gene expression profiles are presented as a heatmap.

To investigate the molecular basis of hyperalgesia associated with OVX and the antinociceptive effect of TPTD, we conducted a transcriptome analysis of RNA sequencing (RNA-seq) in the DRG collected from rats in these experimental groups (Fig. 6). Genes were identified by mapping onto the rat genome, and any gene that had 0 mapped reads for each sample was removed, resulting in 12,742 genes.

The expression of 619 genes differed significantly between the Sham-vehicle group and the OVX-vehicle group, while that of 464 genes differed significantly between the OVX-vehicle group and the OVX-TPTD group (p < 0.05, *t*-test). Among the significantly different genes, 182 and 179 had an absolute log2 (fold change)> 0.25 between the Sham-vehicle group and the OVX-vehicle group and between the OVX-vehicle group and the OVX-TPTD group, respectively.

Differentially expressed genes (DEGs) were functionally annotated and clustered by the Gene Ontology (GO) Biological Processes, Reactome Gene Sets, Kyoto Encyclopedia of Genes and Genomes (KEGG) Pathway, and Comprehensive Resource of Mammalian protein complexes (CORUM) using the Metascape webtool. The top 20 enriched terms or pathways are shown in Supplementary Figure 6a,b by their log10(p-values). The Metascape analysis revealed that the DEGs between the Sham-vehicle group and the OVX-vehicle group (182 genes) were significantly associated with tissue remodeling (GO:0048771) and neuroactive ligand-receptor interaction (KEGG:hsa04080) (-log10(p-values) > 6) (Supplementary Figure 6a), while the DEGs between the OVX-vehicle group and the OVX-TPTD group (179 genes) were significantly associated with cytokine-mediated signaling pathways (GO:0019221) and the regulation of the response to cytokine stimuli (GO:0060759) (-log10(p-values) > 6) (Supplementary Figure 6b). Sixteen genes were commonly identified in these 2 comparisons (Fig. 6, Supplementary Figure 6c).

We next conducted an analysis focused on pain. The genes associated with pain and inflammation were selected from the DEGs mentioned in previous studies on bone pain^3–5^, the Pain Genes Database^28^, and the gene list of Inflammatory Cytokines and Receptors RT Profiler PCR Array. The DEGs associated with pain and inflammation were validated by quantitative real-time polymerase chain reaction (qPCR). In addition, *Calca* (p < 0.05 and log2(fold change) = 0.20, Sham-vehicle vs. OVX-vehicle), which has already been reported to be increased by OVX, was also validated by qPCR.

**Figure 7:**
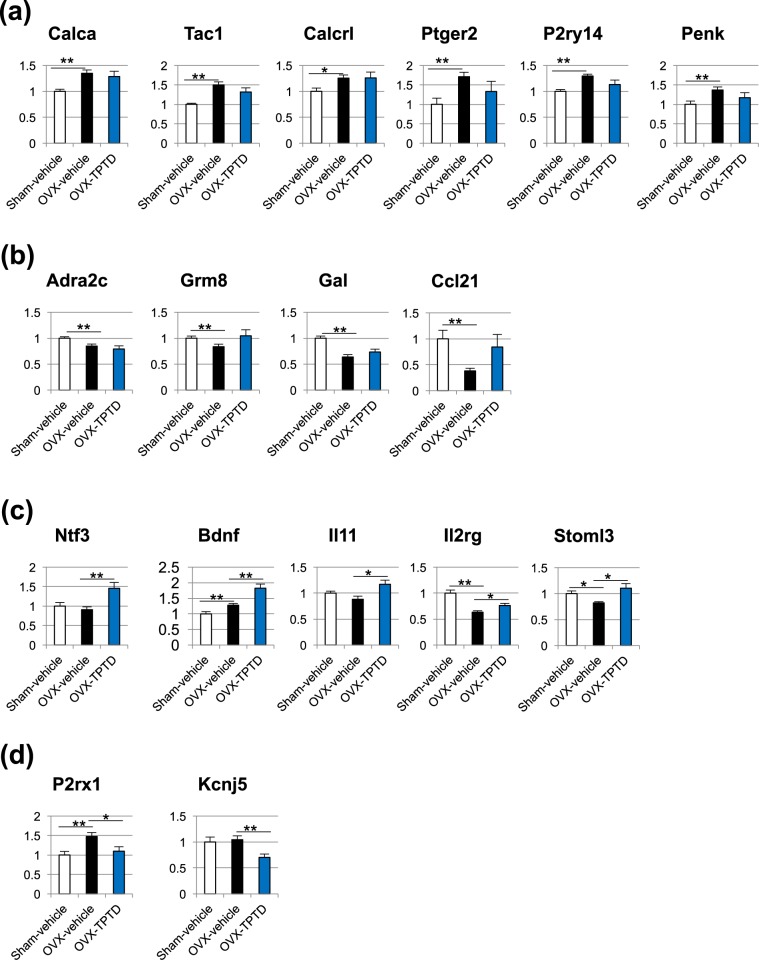
The validation of the changes in the expression of the selected pain- and inflammation-related genes by qPCR. The differentially expressed genes associated with pain and inflammation were validated by qPCR. (**a**) Genes upregulated by OVX: *Calca, Tac1, Calcrl, Ptger2, P2ry14, Penk*; (**b**) genes downregulated by OVX: *Adra2c, Grm8, Gal, Ccl21*; (**c**) genes upregulated by TPTD: *Ntf3, Bdnf, Il11, Il2rg, Stoml3*; (**d**) genes downregulated by TPTD: *P2rx1, Kcnj5*. Significant difference: *p < 0.05, **p < 0.01. Values are normalized against the corresponding expression of *Gapdh* and relative to the Sham-vehicle group, presented as the means + S.E.M.

Seventeen genes were ultimately detected through these analyses (Figs. 6 and 7) and categorized into 2 groups: genes that showed changes in their transcriptional level by OVX (Fig. 7a,b), and genes that showed changes in their transcriptional level by TPTD treatment (Fig. 7c,d). Genes whose transcription was upregulated by OVX included *Calca, Tac1, Calcrl, Ptger2, P2ry14, and Penk* (Fig. 7a), while those that were downregulated included *Adra2c, Grm8, Gal*, and *Ccl21* (Fig. 7b). Genes whose transcription was upregulated by TPTD included *Ntf3, Bdnf, Il11, Il2rg* and *Stoml3* (Fig. 7c), while those that were downregulated included *P2rx1* and *Kcnj5* (Fig. 7d), although the transcription of some of these genes, such as *Bdnf, Il2rg, Stoml3* and *P2rx1*, was significantly altered by OVX.

To functionally annotate and cluster these 17 genes, we conducted the Metascape enrichment analysis as described above. The gene functional annotation obtained by this analysis is shown in a hierarchical clustering heatmap and two-dimensional functional mapping by t-SNE (Supplementary Figure 7a,b, respectively). This analysis allowed us to categorize the 17 genes into functional groups, such as CGRP-related and neuropeptides (*Calca, Calcrl, Gal* and *Tac1*), neurotrophic factors (*Ntf3* and *Bdnf*), interleukin-cytokine related (*Il11, Il2rg* and *Ccl21*), and others.

### Intracellular response of DRG neurons to TPTD

**Figure 8:**
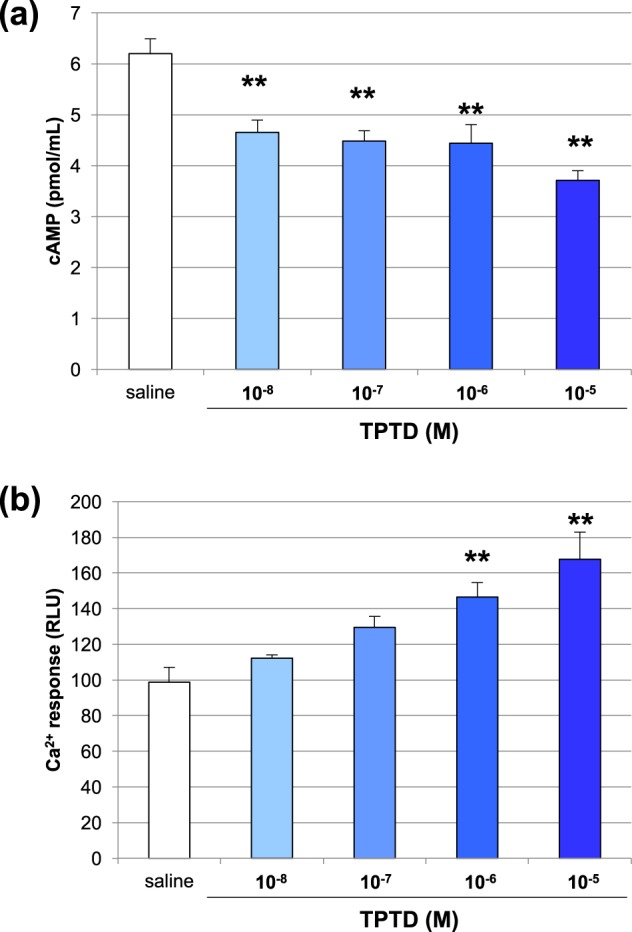
Cellular responses to TPTD administration in cultured DRG neurons. (**a**) The intracellular cAMP level in cultured DRG neurons was significantly reduced by TPTD administration as the TPTD concentration increased. (**b**) TPTD administration in cultured DRG neurons increased the intracellular Ca^2+^ level as the TPTD concentration increased. Significant difference: **p < 0.01.

To determine whether or not TPTD directly induces cellular response in the DRG neurons, we performed *in vitro* experiments using primary neuronal cells cultures obtained from rat DRG (Fig. 8). We measured the cellular levels of cAMP and Ca^2+^, since PTH1R is a G-protein-coupled receptor that regulates these intracellular second messengers upon activation. Treatment of the cultured rat DRG neurons with TPTD significantly decreased the cAMP levels as the TPTD concentration increased (Fig. 8a), whereas the same treatment significantly increased the intracellular Ca^2+^ levels at TPTD doses of 10^−4^ and 10^−5^ M (Fig. 8b). These data suggested that TPTD may transduce intracellular signaling in rat DRG neurons through Gαi and Gαq-coupled but not canonical Gαs signaling mediated by PTH1R in osteoblast-lineage cells (Supplementary Figure 8).

## Discussion

In this study, we applied 2 pain-related behavioral tests—the plantar test (paw flick test, a test observing avoidance of heat stimulation) and the von Frey test (a test to evaluate hyperalgesia by observing avoidance of mechanical stimulation)—to OVX rats to experimentally evaluate whether or not TPTD exerts an antinociceptive effect on this osteoporotic animal model, as has been observed in several clinical reports^15–25^. The plantar test showed that the withdrawal latency was gradually reduced in the OVX rats during the observation period compared with the Sham-vehicle group, confirming thermal hyperalgesia caused by OVX^8,10,11^. The reduced latency significantly recovered in the OVX-TPTD group starting at the initial administration compared to the OVX-vehicle group. The von Frey test also showed that the withdrawal threshold in the OVX-TPTD group was significantly higher than that in the OVX-vehicle group at four weeks after the initial administration. Therefore, we confirmed that administering TPTD to OVX rats resulted in an antinociceptive effect.

This antinociceptive effect was initiated at 6 h after administration in the plantar test. A member of our study team previously reported that the serum level of osteocalcin, an anabolic bone marker, was transiently downregulated at 6 h and then augmented 1 day after the initial TPTD administration^29^. However, in our study, the urinary CTX level was transiently increased at 6 h and then recovered to the basal level on 1 day after TPTD administration. Similar to our own study, the previous study further stated that bone histomorphometric parameters, such as osteoid surface (OS/BS) and osteoblast surface (Ob.S/BS), showed no significant changes at 8 h but did show increases by 2 days after TPTD administration. TPTD also promoted microfracture healing, but only after 2 weeks longer^30^. These findings indicate that the antinociceptive effect is exerted earlier than the bone anabolic effect by TPTD, suggesting that the pharmacological antinociceptive action of TPTD is independent of its effect on bone metabolism.

To substantiate the TPTD effect on the pain-related behavioral tests, we next conducted morphometrical analysis of Iba-1-positive microglia in the spinal cord. Activation of spinal microglial cells has been implicated in nerve injury-induced neuropathic pain, which is characterized by proliferation and morphological hypertrophy of these cells and enhanced expression of microglial molecules^26,27,31,32^. Our morphometrical analyses on spinal microglial cells demonstrated that the OVX-induced hyperalgesia involved the increased number and hypertrophy of microglia as has been observed in neuropathic pain models. As far as we know, this is the first evidence that microglial reactivity is involved in OVX-induced hyperalgesia. TPTD treatment significantly, but not fully, reduced the increased number of spinal microglia, and did not significantly rescued OVX-induced microglial hypertrophy. These findings on spinal microglia appears to be consistent with the observations by the pain-related behavioral tests.

TPTD (the 1–34 fragment of human PTH) reportedly activates its cellular action effectively through rat PTH1R but poorly through rat PTH2R^33,34^. Therefore, in rats, TPTD exerts its pharmacological effects through its binding to PTH1R. Immunofluorescence against PTH1R as well as PTH2R demonstrated that most neurons in the DRG expressed substantial levels of these receptors. The types of sensory neurons that innervate the bone are unmyelinated C-fibers and thinly myelinated Aδ-fibers, and substance P and CGRP-positive nerve fibers have been identified in bone marrow, bone cortex, and periosteum^4,35^. The populations of C-fibers and Aδ-fibers in the DRG contains 30–40% of CGRP-positive neurons. In this study, we noticed 2 types of CGRP-positive neuronal cell bodies in the DRG, relatively small cells with low CGRP level, and large cells with high CGRP level. These are possibly subpopulations of C-fibers and Aδ-fibers, respectively. Therefore, PTH receptors were expressed regardless of the type of nerve fibers. In a classical scheme, hyperalgesia toward both heat stimulation and mechanical stimulation is attributed to the involvement of C-fibers, while Aδ-fibers mainly transmit mechanical stimulation, although such fiber-specific function is currently controversial^36,37^. In this study, TPTD exhibited approximately the same antinociceptive effect in both the plantar test and von Frey test. Therefore, these behavior tests, along with our immunofluorescence analyses, suggested no fiber-specific prevalence of TPTD-induced neuronal effects.

The treatment of cultured rat DRG neurons with TPTD significantly decreased the cAMP levels and increased the Ca^2+^ levels as the TPTD concentration increased. These data suggested that PTH1R could deliver signals through a Gq and Gi-coupled downstream pathway in rat DRG neurons. PTH treatment of cells expressing the Na^+^/H^+^ exchange regulatory cofactor 2 (NHERF2)–PTH1R complex was reported to markedly activate phospholipase C and inhibit adenylyl cyclase through the stimulation of Gi protein^38^. This finding suggests that NHERF2 plays a critical role in neuronal signaling but not in osteoblastic signaling by TPTD. Consistently, NHERF2 was not detected in ROS 17/2.8 cells, a rat osteoblastic line^38^. Recent work reported that activation of PTHrP/PTH1R mediated signaling in cultured human DRGs was suggested to be involved in peripheral heat and mechanical hypersensitivity^39^, suggesting opposite neural function of PTHrP and PTH although they share the same receptor; PTH1R. Their binding preferences to different conformations of PTH1R may cause distinct neuronal function as proposed in osteoblast lineage cells^40^.

To understand the molecular basis of OVX-induced hyperalgesia and the antinociceptive effects of TPTD, this study investigated the functional changes in primary neurons. A transcriptome analysis by RNA-seq and successive bioinformatics showed that changes in the transcriptomal profile were associated with OVX-induced hyperalgesia and the antinociceptive effects of TPTD. On comparing the Sham-vehicle and OVX-vehicle groups, clustering of 182 significantly changed genes revealed a high association with tissue remodeling and neuroactive ligand-receptor interaction (Supplementary Figure 6a). The same comparative analysis of 179 significantly changed genes between the OVX-vehicle and OVX-TPTD groups showed a high association with the cytokine-mediated signaling pathway and the regulation of the response to cytokine stimuli (Supplementary Figure 6b). These analyses suggest that the antinociceptive effect induced by TPTD in OVX rats involved functional changes in the cytokine pathway and cellular response to cytokines in primary sensory neurons and that TPTD did not directly restore the changes in the transcriptomal profile induced by OVX in these neurons.

We next analyzed the 17 genes that were validated by qPCR. Functional annotation and clustering divided these 17 genes into groups of CGRP-related and neuropeptides, neurotrophic factors, interleukin-cytokine related genes, and others (Supplementary Figure 7). The CGRP-related and neuropeptides included *Calca, Calcrl, Tac1*, and *Gal. Calca, Calcrl*, and *Tac1* were significantly upregulated in the OVX-vehicle group compared to the Sham-vehicle control group. It was reported that the expression of CGRP (*Calca*) and substance P (*Tac1*) in the DRG was increased by OVX. CGRP and substance P have been implicated in OVX-induced hyperalgesia^41–43^. CGRP reportedly induces hyperalgesia via the CGRP receptor, so elevated CGRP expression is suggested to produce pain^44^. Changes in the expression of CGRP and substance P by OVX were also observed in this study. Calcitonin receptor-like receptor (CLR; *Calcrl*) is a G protein-coupled receptor for CGRP. The upregulation of *Calcrl* in the DRG was reportedly involved in a rat model of osteoarthritis pain^45^. Therefore, these previous findings are consistent with our results and validate our analyses of animal experiments and bioinformatics. Interestingly, in this gene group, *Gal* was the only gene that was significantly downregulated in the OVX-vehicle group. *Gal* encodes two mature peptides: galanin and galanin message-associated peptide (GMAP). *Gal*-deficient mice exhibited developmental and regenerative deficits in the DRG neurons^46^. Galanin has been reported to be involved in nociception associated with inflammatory pain^47^. These findings and our analysis suggest the mechanistic involvement of neuronal tissue damage in this skeletal pain.

The other genes that were upregulated in the OVX-vehicle group compared to the Sham-vehicle control group were *Ptger2* (encoding prostaglandin E receptor 2), *P2ry14* (purinergic receptor P2Y14) and *Penk* (Proenkephalin), all of which reportedly function in the DRG neurons and are associated with inflammatory pain and neuropathic pain^48–50^. The genes that were significantly downregulated other than *Gal* in the OVX-vehicle group included *Adra2c*, *Grm8*, and *Ccl21*. *Adra2c* encodes an alpha-2 adrenergic receptor that regulates neuronal transmission in the terminal of the DRG neurons. Therefore, the downregulation of *Adra2c* in the DRG neurons by OVX may affect the descending pain modulatory system. *Grm8* encodes the group III metabotropic glutamate receptor that negatively modulates transient receptor potential cation channel subfamily V member 1 (TRPV1) through the inhibition of adenyl cyclase and downstream intracellular activity, thus blocking the TRPV1-induced activation of nociceptors^51^. The downregulation of *Grm8* may cause the hyper-activation of nociceptors. *Ccl21* encodes the chemokine ligand CCL12, whose upregulation is reportedly involved in neuropathic pain development^52^. Taken together, the findings from our analyses suggest that the pathogenesis of OVX-induced hyperalgesia involves the hyper-transduction of pain signals and impairment of the descending pain modulatory system as well as neuropathic tissue damage.

Given the molecular basis of the antinociceptive effect of TPTD administration to OVX rats, this pharmacological effect in the DRG neurons involved significant changes in the expression of genes that were functionally categorized into neurotrophic factors, such as *Ntf3* and *Bdnf*, and interleukin-cytokine-related factors, such as *Il11* and *Il2rg. Ntf3* and *Bdnf* encode neurotrophin-3 and brain-derived neurotrophic factor (BDNF), respectively. These factors reportedly function in DRG neurons and are associated with inflammatory and neuropathic pain^53,54^. These neurotrophic factors help support the survival of existing neurons and encourage the growth and differentiation of new neurons and synapses. However, BDNF is also induced in spinal microglia as well as in the DRG after nerve injury, which possibly contribute to pain transmitting dorsal horn neurons by modulating the neuronal activity^27,32^. It is, therefore, suggested that further upregulation of BDNF by TPTD is associated with its partial rescue of the pain-related behavioral and the microglial reactivity in the spinal cord. *Il11* encodes interleukin (IL)-11, a cytokine belonging to the IL-6 family, which has anti-inflammation properties. It was reported that treatment with IL-11 resulted in an anti-inflammatory effect in collagen-induced arthritis model mice^55^. IL-11, also referred to as a neuropoietic cytokine, influences the DRG neuron survival^56^. *Il2rg* encodes IL2RG, a component of the receptors for IL-2, IL-4, IL-7, IL-9, and IL-15. Of note, it was reported that TPTD treatment enhanced the *Il11* expression though protein kinase C (PKC) in osteoblasts^57^, suggesting a direct target molecule by PTH-mediated signaling, even in the DRG neurons. Therefore, the antinociceptive effect by TPTD is suggested to be exerted partly through the induction of factors that may contribute to neuronal tissue repair in the wake of neuropathic tissue damage induced by OVX.

*Stoml3* was downregulated by OVX and upregulated by TPTD administration in OVX rats in our study. *Stoml3* encodes stomatin-like protein-3 (STOML3), which modulates mechanotransduction channels and acid-sensing ion channels (ASICs). STOML3 plays important roles in several pain models; however, its function in pain seems to be paradoxical^58–60^. *P2rx1* and *Kcnj5* were significantly downregulated in the OVX-TPTD group. *P2rx1* encodes the purinergic receptor P2X1, and this gene expression was reported to be upregulated in the DRG neurons in a neuropathic pain model^61^ as well as in the OVX rats in the present study. *Kcnj5* encodes G protein-activated inward rectifier potassium channel 4. *Kcnj5-*deficient mice exhibit hyperalgesia^62^. Therefore, the functional relevance of these genes in the antinociceptive effect by TPTD should be further investigated.

It has been proposed that a possible mechanism of osteoporotic pain is chronic neuronal excitation in intraosseous sensory nerve systems by acids and activated inflammatory cytokines in bone marrow environment^3,4^. Using a mouse OVX model, Dhoke *et al*. recently demonstrated that the antinociceptive effect by TPTD was associated with the downregulation of inflammatory cytokine expression, including IL-1β, IL-6 and TNF-α in bone tissue^63^. Osteoporotic pain involves hypoactivity of the descending inhibitory nerve system in the spinal cord that is associated with the decreased expression of serotonin receptors^3,7^. Therefore, it is will be relevant to unveil whether the pharmacological regulation in the DRG neurons of PTH is associated with the changes in the bone marrow environment and the descending inhibitory nerve system. It will be also interesting to investigate how these molecules discussed above are participated in activation of spinal microglia, since injured sensory neuron in DRG can release molecular signal to the dorsal horn by intra-axonal transport, which directly enables communication between DRG neurons and spinal microglia^32,64^.

Our study demonstrated that TPTD administration immediately induced an antinociceptive effect in OVX rats, a timepoint that was earlier than that noted with its bone anabolic effects. This antinociceptive effect by TPTD was partial, and possibly exerted partly through changes in the expression of genes that are associated with neuronal tissue repair. The DRG neurons expressed PTH1R, whereas TPTD mediated unique Gi and Gq signaling via PTH receptor in the cultured DRG neurons, suggesting a direct target of PTH (Supplementary Figure 8). In conclusion, non-canonical PTH signaling in primary sensory neurons was suggested to contribute to the antinociceptive effect by TPTD in OVX rats, which involves changes in neuro-protective and inflammatory genes that can regulate the reactivity of spinal microglia.

## Materials and methods

### Experimental animals

Ten-week-old female Sprague-Dawley rats were used for this study (Charles River, Kanagawa, Japan). The rats were maintained under light and dark cycles (12–12 h) and allowed to unrestricted access to tap water and food (CRF-1; Oriental Yeast, Tokyo, Japan)^65^. Cages were enriched with nesting material (Enviro-dri; Shepherd Specialty Papers, Watertown, TN, USA). The rats were allowed to acclimate to their environment for 10 days before the start of the experiments. The experimental study was approved by the Experimental Animal Ethics Committee at LSI Medience Corporation and Asahi Kasei Pharma Corporation and performed according to established guidelines for the management and handling of experimental animals.

### Experimental design

Twelve-week-old rats were randomized by withdrawal latency in the plantar test and body weight into 2 groups: the OVX group (n = 20) and the Sham group (n = 10). Bilateral OVX or sham surgery was performed under anesthesia. In the sham surgery, the ovaries were exteriorized but not removed. Four weeks after surgery, the OVX group rats were further randomized into 2 groups: the OVX-vehicle group (n = 8) and the OVX-TPTD group (n = 8). Eight specimens in each group were selected by the average body weight and the results of the preceding plantar test (described in a following section) and subjected to further analyses. Accordingly, 8 rats close to the average latency in the plantar test were selected from the Sham group (n = 10) and defined as the Sham-vehicle group. The rats received the following treatments for 4 weeks: subcutaneous administration of TPTD (Asahi Kasei Pharma, Tokyo, Japan) at a dose of 30 μg/kg body weight 3 times a week (OVX-TPTD group) or subcutaneous administration of saline 3 times a week (Sham-vehicle and OVX-vehicle groups). All rats were sacrificed by exsanguination from the abdominal aorta under isoflurane anesthesia at the end of the experimental period. The fourth and fifth lumbar DRG (L4–5 DRG), right femur, and blood samples were then collected for further analyses (Fig. 1). Two pain-related behavioral tests were performed as described below.

### Pain-related behavioral test

#### Plantar test

Rats were allowed at least two 30-min sessions to habituate to the test environment prior to behavioral testing. Thermal hypersensitivity was tested according to the Hargreaves procedure^66^ using an analgesia meter (Plantar test 7370; Ugo Basile, Varese, Italy).

In brief, rats were placed in clear plastic boxes and allowed to acclimate. A constant-intensity radiant heat source was directed to the midplantar area of the left hind paw. The time from activation of the heat source until paw withdrawal was recorded as the paw withdrawal latency. The cut-off time was set for 15 seconds to avoid tissue damage. The plantar tests were conducted before surgery and 1, 2, 3, and 4 weeks after surgery to monitor hyperalgesia induced by OVX. After initial TPTD administration, the hypersensitivity was tested on the day of the first and second administration and every week to evaluate the effect on hyperalgesia. The plantar tests were conducted 5–6 h after the TPTD administration.

#### von Frey test

Rats were allowed at least two 30-min sessions to habituate to the test environment prior to behavioral testing. Mechanical sensitivity was measured using von Frey monofilaments on the left hind paw at the day of the final TPTD administration (5–6 h after administration). The von Frey withdrawal threshold was determined by adjusting the stimulus intensity between 1.0 and 26.0 g equivalents of force according to the up-and-down method^67^.

In brief, rats were placed on mesh platforms in a plastic chamber. After acclimation for more than 5 min, filaments of sequentially increasing stiffness with initial bending force of 4.0 g were applied to the hind paw. A positive response was defined as the withdrawal of the hind paw to the stimulus. The withdrawal threshold was determined by sequentially increasing and decreasing the stimulus intensity.

### Skeletal analyses

#### Measurement of the bone mineral density (BMD)

The right femur samples were collected and stored at −80 °C until analyses. Before measurement, the femur samples were immersed in saline at room temperature and removed the attached soft tissue. The BMD of the femur was measured using dual energy X-ray absorptiometry (DXA) equipment (DCS-600EX-3R; Aloka, Tokyo, Japan). The femur samples were placed on the scan table and scanned at a pitch of 1 mm and speed of 25 mm/min. The BMD (mg/cm^2^) was calculated from the bone mineral content (mg) and bone area (cm^2^)^65^. For the analysis, the femur was divided into three regions (proximal, shaft, and distal), and the BMD in each region was obtained.

#### Measurement of bone metabolic markers in serum samples

All rats were fasted for at least 6 h before sacrificing and blood sampling. Serum samples were obtained by centrifugation of the blood samples. The serum samples were aliquoted and stored at −80 °C until analyses. The level of osteocalcin, a bone formation marker, was determined using a rat osteocalcin ELISA kit(GE Healthcare Bioscience, Piscataway, NJ, USA). The level of C-terminal telopeptide of type I collagen (CTX), a bone resorption marker, was measured using a RatLaps ELISA kit (Nordic Bioscience Diagnostics, Copenhagen, Denmark). Both assays were performed according to the manufacturers’ instructions.

### Gene expression analyses

#### RNA isolation

Rats were sacrificed under isoflurane anesthesia, and the L4–5 DRG was readily dissected. The tissue samples were then immersed in 0.2 mL RNAlater (Thermo Fisher Scientific, Waltham, MA, USA) and stored at −80 °C until use. Total RNA from each sample was isolated using a RNeasy lipid-tissue mini kit with DNase I (QIAGEN, Hilden, Germany) to reduce contamination of genomic DNA prior to further analyses.

#### RNA sequencing (RNA-seq)

Sequence libraries were prepared using the TruSeq Stranded mRNA Sample Prep Kit (Illumina, San Diego, CA, USA). Sequencing was performed in the paired-end (100 ×2) mode on the HiSeq. 2500 (Illumina) using a TruSeq SBS Kit v4-HS (Illumina). Sequenced reads data were mapped onto the *Rattus norvegicus* genome (rn6) using the TopHat software program v2.0.14 based on the Bowtie v2.2.5 and SAMtools v1.2 software programs. Fragments per kilobase per million (FPKM) values were computed by the Genedata Expressionist software program v9.1.4a (Genedata, Basel, Switzerland) (performed by Takara Bio, Shiga, Japan).

Metascape (http://metascape.org/) was used for the gene set enrichment analysis and functional annotation. A Metascape analysis is carried out with four sources: GO Biological Processes, Reactome Gene Sets, KEGG Pathway, and CORUM. All analyses were performed with converting the input rat genes into their human orthologs as suggested by the Metascape manual.

We referred to the literature concerning bone pain^3–5^, the Pain Genes Database^28^ (http://www.jbldesign.com/jmogil/enter.html), and the gene list of Inflammatory Cytokines and Receptors RT Profiler PCR Array (https://www.qiagen.com/us/shop/pcr/primer-sets/rt2-profiler-pcr-arrays/?catno=PARN-150Z#geneglobe) as the list of the genes associated with pain and inflammation. The gene expression profiles were presented as a heatmap using the gplots package in R v3.4.1. Hierarchical clustering of the gene expression was performed with Euclidean distance and the group average method.

The gene functional annotation profiles were presented as a heatmap based on the Metascape annotation data also using the gplots package in R. Similarly, hierarchical clustering of the gene functional annotation was performed with Euclidean distance and the group average method. Two-dimensional functional mapping was performed with t-distributed stochastic neighbor embedding (t-SNE) using the Rtsne package (perplexity = 5) in R.

#### Quantitative real-time polymerase chain reaction (qPCR)

Isolated total RNA was subjected to cDNA synthesis with random primers using the SuperScript VILO cDNA Synthesis Kit (Thermo Fisher Scientific). The synthesized cDNA was used as templates for qPCR using the TaqMan Gene Expression Master Mix Kit and Taqman Gene Expression Assay (Thermo Fisher Scientific) on a QuantStudio 7 Flex System (Applied Biosystems, Waltham, MA, USA). Data were normalized against the corresponding expression levels of *Gapdh*. The TaqMan Gene Expression assays used in this study are listed in Supplementary Table 1.

### Immunohistochemistry, microscopic systems and fluorescence morphometry

Rats were anesthetized and sacrificed via exsanguination from the abdominal aorta. The L5 spinal cord segments and the lumbar vertebrae (L5) including the DRG were dissected and fixed in 4% paraformaldehyde at 4 °C for 2 days. Decalcifications of the lumbar vertebrae samples were carried out in 10% EDTA-2Na solution (Muto Pure Chemicals, Tokyo, Japan) for 2 weeks at 4 °C. After paraffin embedding, paraffin tissue blocks were cut into 5-μm-thick slices. Paraffin-embedded sections were de-paraffinized, and epitope retrieval was performed using a rice steamer at 95 °C for 30 min in 10 mM citrate buffer. The tissue sections were incubated in 5% bovine serum albumin (BSA) (Sigma-Aldrich, St. Louis, MO, USA) in Tris-buffered saline with Tween 20 (TBS-T) (Takara Bio) for 30 min at room temperature and then washed. The samples were then incubated at 4 °C for 15 h in anti-Iba-1 (1:1000; Wako, Osaka, Japan), anti-PTH1R (1:200; Merck Millipore, Burlington, MA, USA), anti-CGRP (1:200; Enzo Life Sciences, Farmingdale, NY, USA), anti-NF200 (1:200; Sigma-Aldrich), or anti-PTH2R (1:200; Thermo Fisher Scientific). After being washed, the slides were incubated with anti-mouse IgG Alexa Fluor 488 (1:200; Thermo Fisher Scientific), anti-rabbit IgG Alexa Fluor 568 (1:200, Thermo Fisher Scientific), and DAPI (1:1000; Dojindo Laboratories, Kumamoto, Japan) for 1 h, followed by final washing. Finally, the slides were mounted with coverslips using Prolong Gold antifade mounting medium (Thermo Fisher Scientific). The same sections were then stained using the Klüver–Barrera method, which enables the simultaneous observation of neuronal cell bodies and myelinated fibers.

Bright-field and fluorescence images of the tissue sections were obtained by a vertical microscopy system with differential interference contrast, an ECLIPSE Ni (Nikon, Tokyo, Japan) equipped with objectives (Nikon), as follows: Plan Apo λ ×10 (numerical aperture [NA] = 0.45), Plan Apo λ × 20 (NA = 0.75), and Plan Apo λ ×40 (NA = 0.95). The fluorescence signals were obtained using the following filter sets: GFP-B (excitation: 460–500 nm, dichroic mirror (DM): 505 nm, emission: 510–560 nm; Nikon), TxRed (excitation: 540–580 nm, DM: 595 nm, emission: 600–660 nm; Nikon), and DAPI (excitation:340–380 nm, DM: 400 nm, emission: 435–485 nm; Nikon). Tiling fluorescence imaging to acquire the entire, high-contrast view of the tissue sections was carried out using a Plan Apo λ × 10 objective (NA = 0.45). The frame size of a single scan was 1280 × 1024 pixels with 8-bit color depth. The fluorescence and differential interference contrast (DIC) images were sequentially acquired, with a pixel size of 0.64 μm. Image processing, including deconvolution, was performed using the imaging software program NIS-elements AR (Nikon).

Fluorescence morphometry of Iba-1-positive cells was carried out NIS-Elements software program (Nikon, Tokyo, Japan). Whole tissue sections from every specimen from the 3 experimental groups were imaged. Regions of interest (ROI) of 400 μm × 400 μm was set in the dorsal horn region for morphometrical analyses. Thresholding was carried out based on frequency profiles of fluorescence intensities through filters of TxRed and DAPI for Iba-1 and DAPI signals, respectively. Object sizes were filtered by 2 μm and 10 μm for DAPI-positive cellular nuclei, and Iba-1-positive microglial clusters, respectively.

### *In vitro* analyses

#### Primary culture of rat DRG sensory neurons

DRGs were isolated from neonatal rats and digested in 1.25 mg/mL collagenase from *Clostridium histolyticum* (Sigma-Aldrich) and 0.2 mg/mL Deoxyribonuclease I from bovine pancreas (Sigma-Aldrich) in Ham’s F-12 Nutrient Mix, GlutaMAX (F-12) (Thermo Fisher Scientific) for 45 min at 37 °C. Dissociated cells were spun through a 10% BSA cushion to remove debris. DRG neurons were plated onto poly-D-lysine-coated 96-well plates at a density of 50,000 cells per well and grown in F-12 supplemented with 1% fetal bovine serum (FBS) (Thermo Fisher Scientific) and 1% Penicillin-Streptomycin (PCSM) (Thermo Fisher Scientific). After overnight culture, the medium was replaced with Neuro basal medium (Thermo Fisher Scientific) supplemented with B-27 (Thermo Fisher Scientific), GlutaMax (Thermo Fisher Scientific), 100 ng/mL nerve growth factor (NGF)-β from rat (Sigma-Aldrich), and 1% PCSM. The experiments were performed after three days of culture.

### Measurements of cyclic AMP (cAMP) production and [Ca^2+^] in DRG sensory neurons

Cultured DRG neurons were rinsed twice in Hank’s Balanced Salt Solution (HBSS) containing 20 mM HEPES and 100 μM 3-Isobutyl-1-methylxanthine (IBMX) and then stimulated with TPTD or vehicle for 15 min at 37 °C. cAMP levels were determined with the Cyclic AMP EIA kit (Cayman Chemical, Ann Arbor, MI, USA) and VersaMax microplate reader (Molecular Devices, San Jose, CA).

Cultured DRG neurons were stained with a FLIPR Calcium-5 Assay Kit (Molecular Devices). TPTD or vehicle was then added to the wells, which were imaged with a FLIPR Tetra System (Molecular Devices) for 400 seconds.

### Statistical analyses

All data are presented as the mean ± standard error of the mean.

The time-course data of plantar test in all groups is analyzed using a two-way ANOVA comparing with the differences among groups and time points. The OVX-vehicle group was compared with the Sham-vehicle group, and group differences were analyzed using Student’s *t*-test at each time point. The OVX-TPTD group was compared with the OVX-vehicle group, and group differences were analyzed using Student’s *t*-test at each time point after the confirmation of a significant difference by two-way ANOVA.

In von Frey test, skeletal analysis, gene expression analyses, and fluorescence morphometry, the OVX-vehicle group was compared with the Sham-vehicle group, and group differences were analyzed using Student’s *t*-test. The OVX-TPTD group was compared with the OVX-vehicle group, and group differences were analyzed using Student’s *t*-test.

In *in vitro* analyses, the effects of TPTD were investigated using Dunnett’s test.

All p values of <0.05 were considered statistically significant.

## Supplementary information

Supplementary Figures and Table.

## Data Availability

The RNA-seq data are deposited at Gene Expression Omnibus (GEO, Accession Number: GSE121109). All other data available from the authors upon reasonable request.
