# Supplementary material for: Teriparatide relieves ovariectomy-induced hyperalgesia in rats, suggesting the involvement of functional regulation in primary sensory neurons by PTH-mediated signaling
Source: Sci Rep. 2020 Mar 24;10:5346. doi: 10.1038/s41598-020-62045-4 (PMC7093455; doi:10.1038/s41598-020-62045-4)
Supplement: Supplementary file 1 — Supplementary Figures and Table. [file 41598_2020_62045_MOESM1_ESM.pdf]

Teriparatide relieves ovariectomy-induced hyperalgesia in rats, suggesting the involvement of functional regulation in primary sensory neurons by PTH-mediated signaling

Tomoya Tanaka[1,2], Ryoko Takao-Kawabata[1*], Aya Takakura[1,2], Yukari Shimazu[1], Momoko Nakatsugawa[1], Akitoshi Ito[1], Ji-Won Lee[3], Koh Kawasaki[1], Tadahiro Iimura[2,3*]

[1]Pharmaceuticals Research Center, Asahi Kasei Pharma Corporation, 632-1 Mifuku, Izunokuni city, Shizuoka 410-2321, Japan

[2]Department of Pharmacology, Graduate School of Dental Medicine, Hokkaido University, N13 W7, Sapporo 060-8586, Japan

[3]Division of Bio-Imaging, Proteo-Science Center (PROS), Ehime University, Shitsukawa, Toon city, Ehime 791-0295, Japan

Supplementary Figure 1

(a)

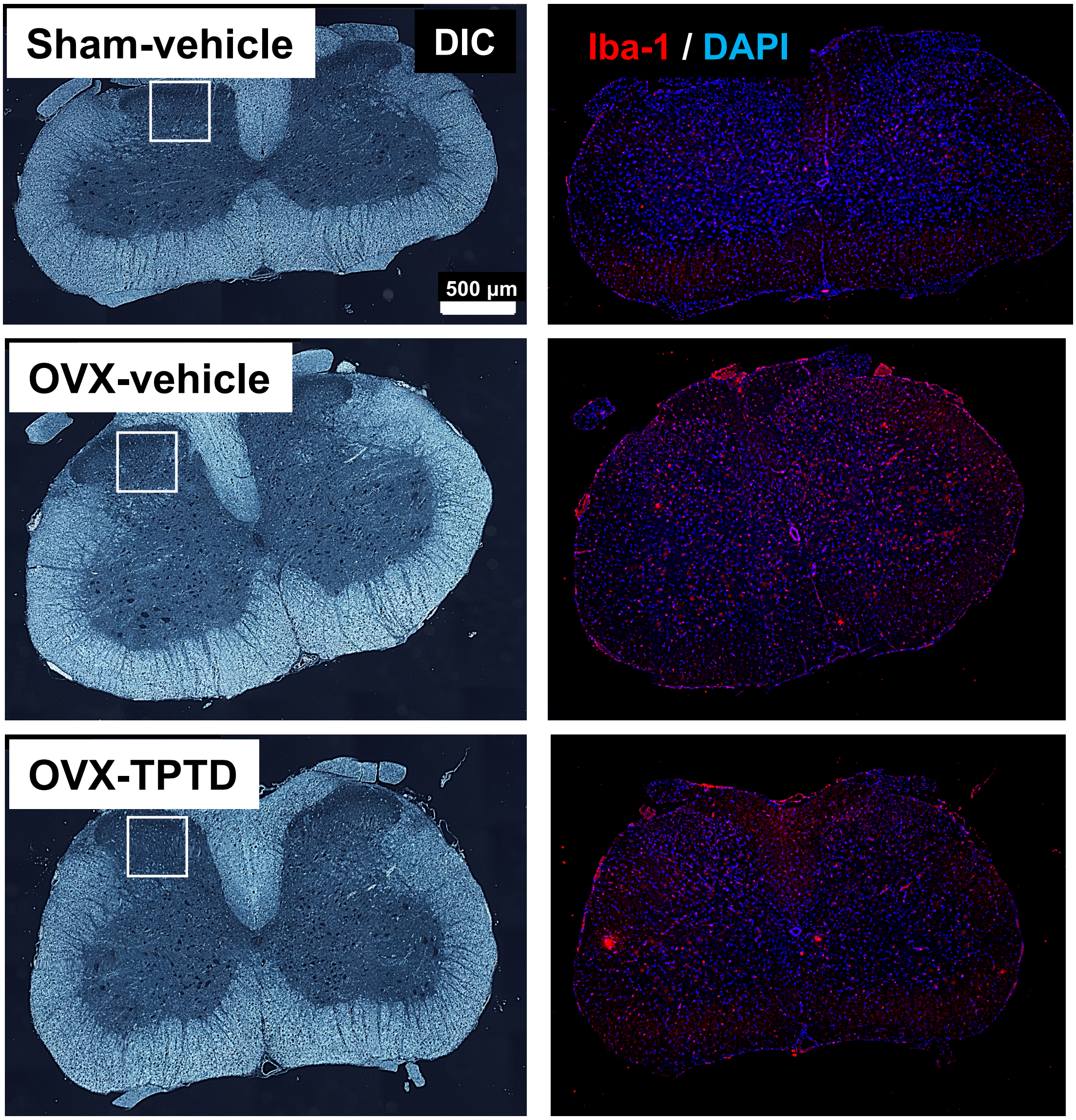

(b)

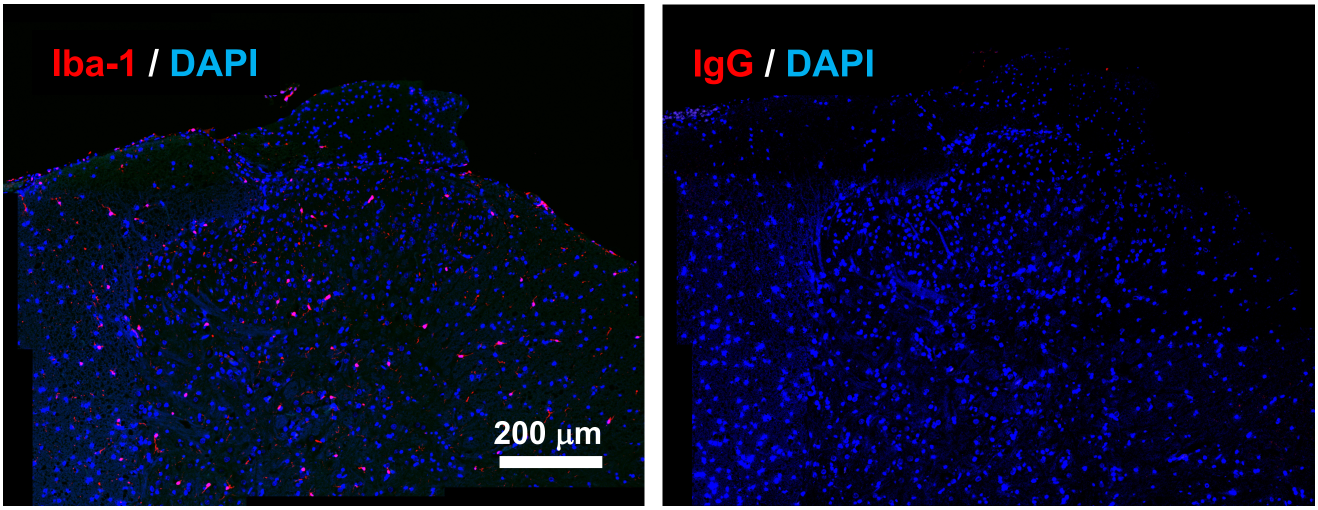

Supplementary Figure 2

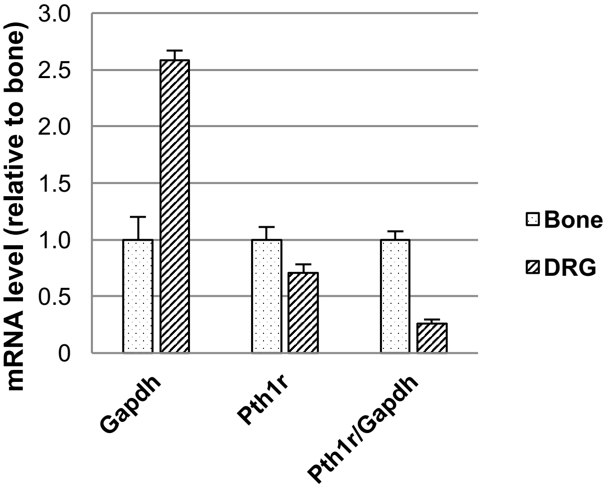

Supplementary Figure 3

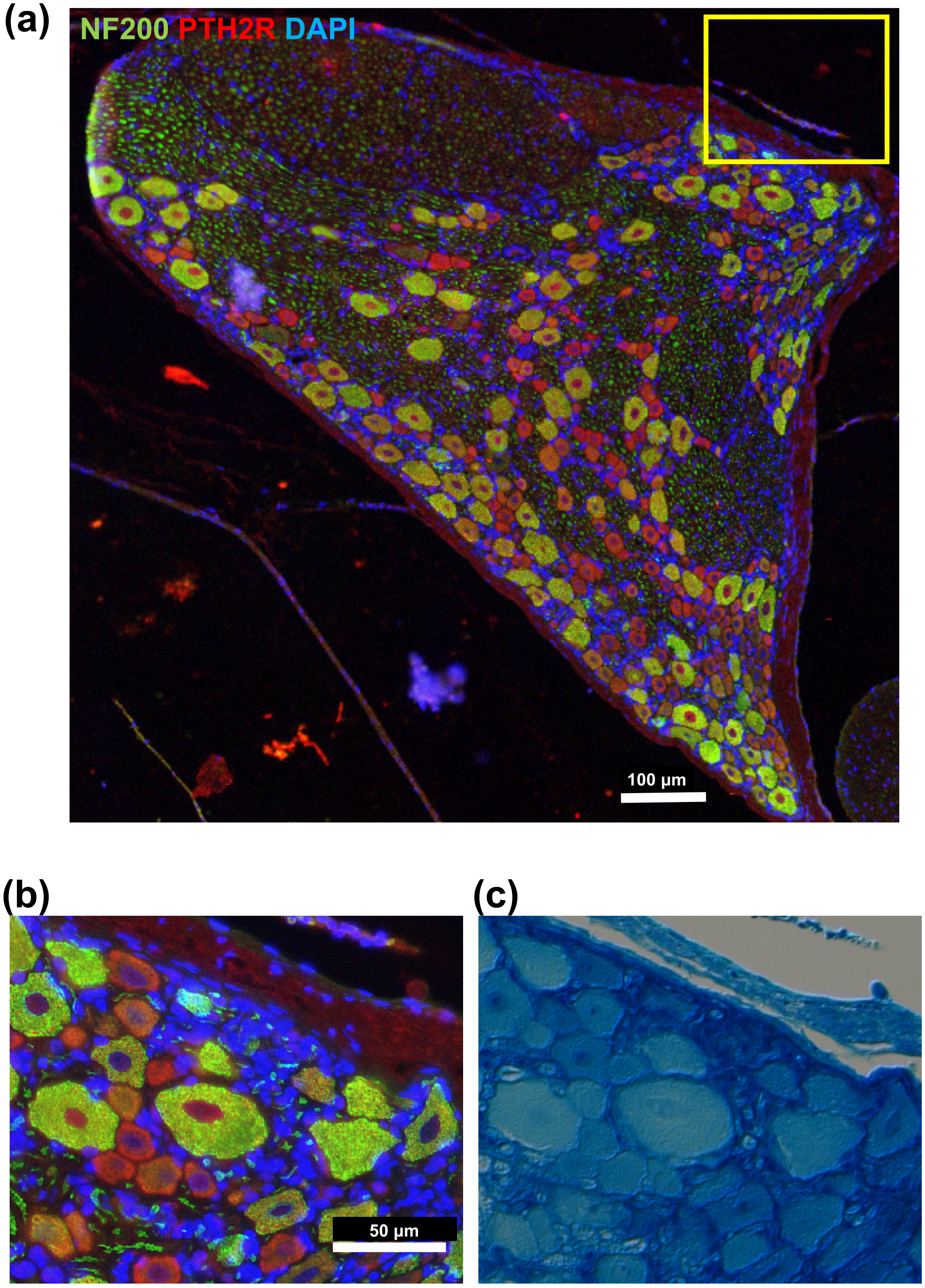

Supplementary Figure 4

(a)

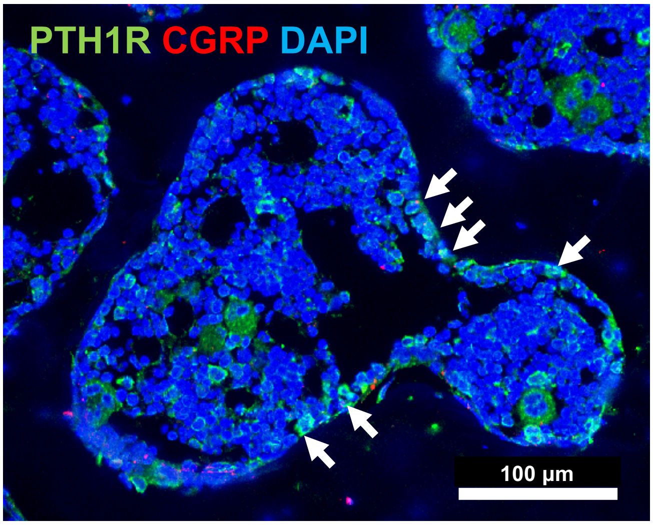

(b)

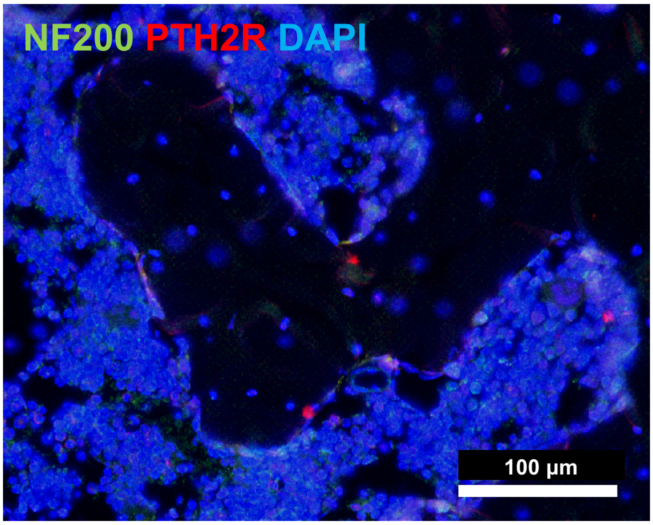

Supplementary Figure 5

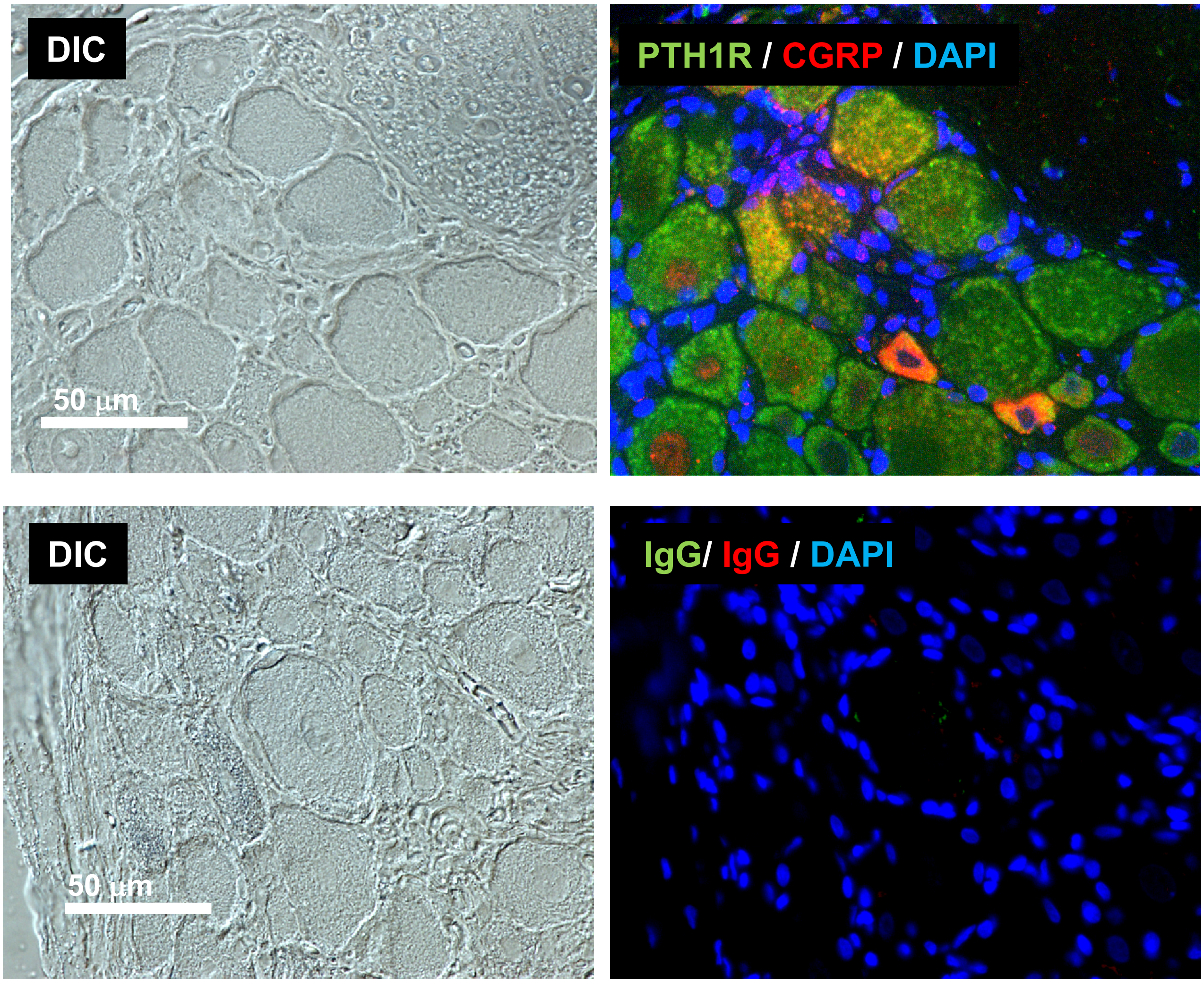

Supplementary Figure 6

(a)

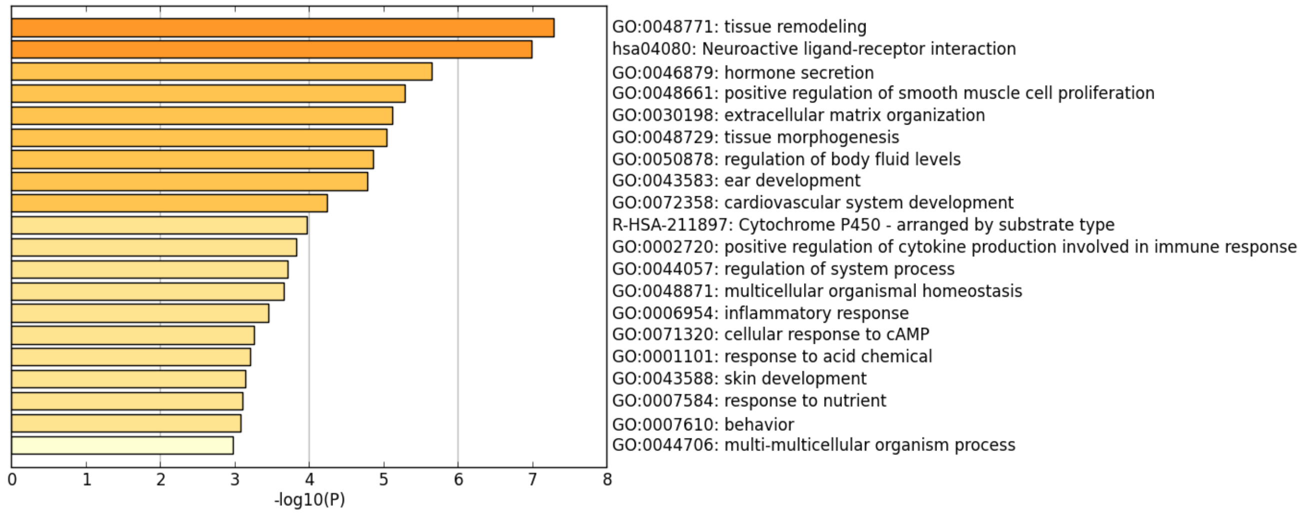

(b)

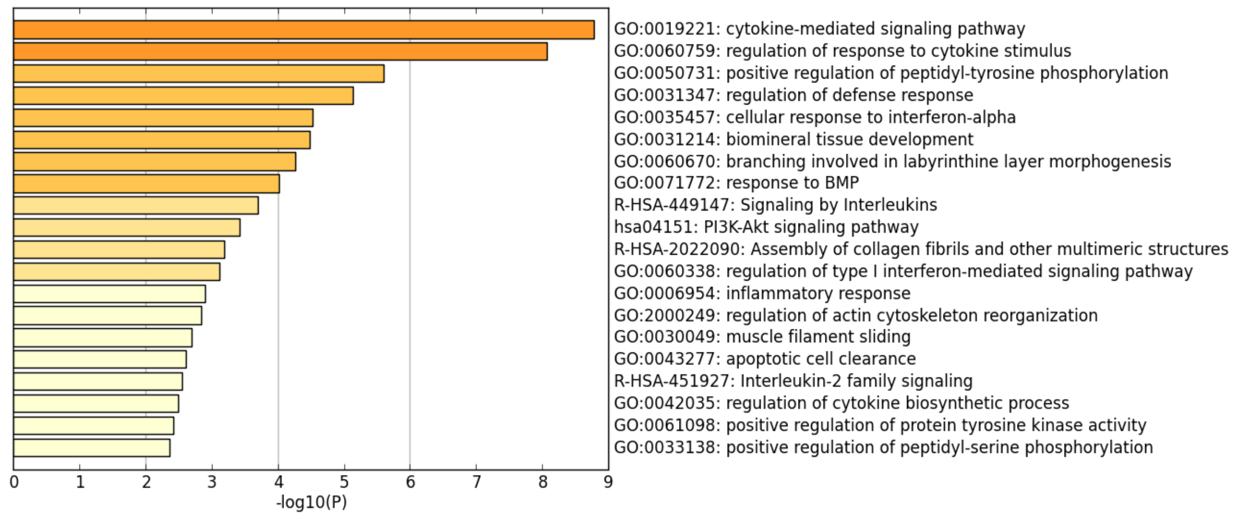

(c)

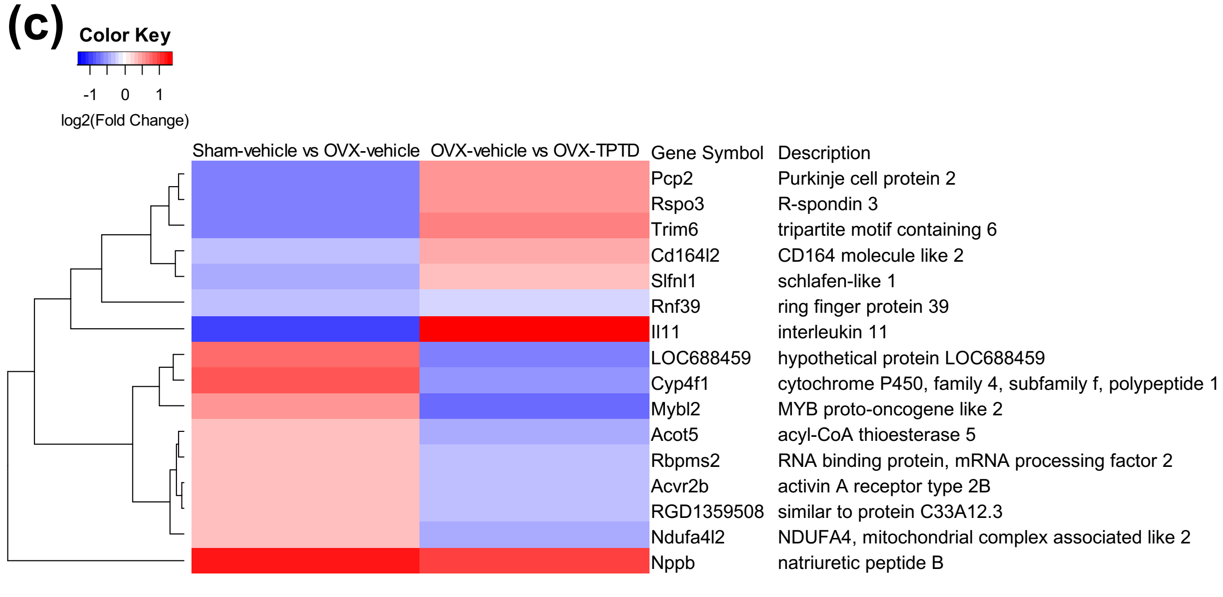

Supplementary Figure 7

(a)

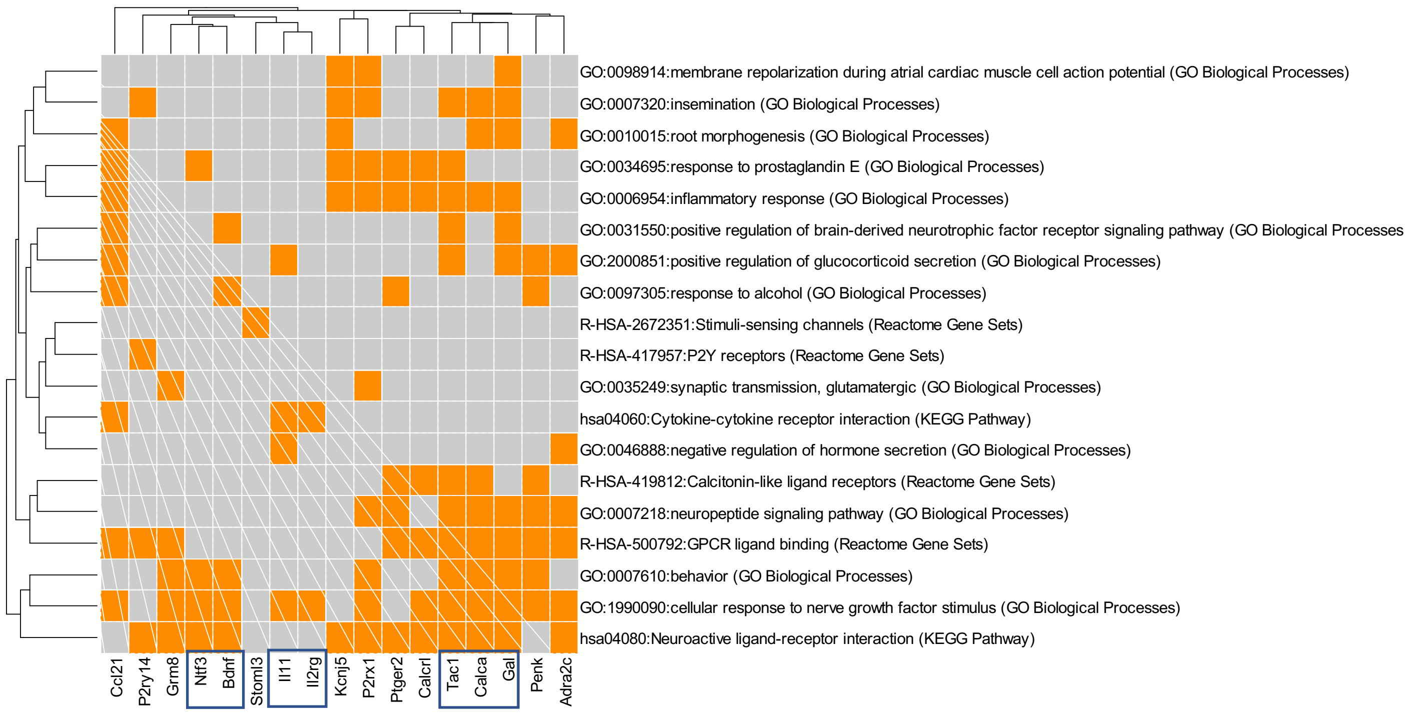

(b)

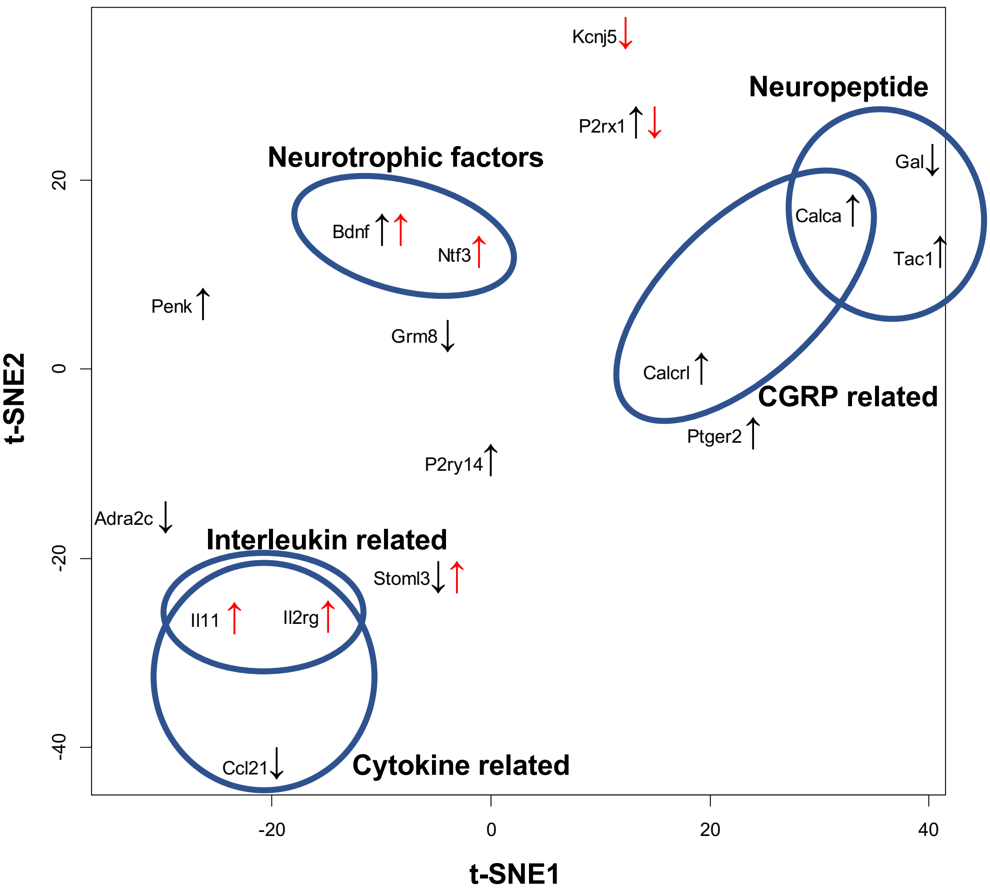

Supplementary Figure 8

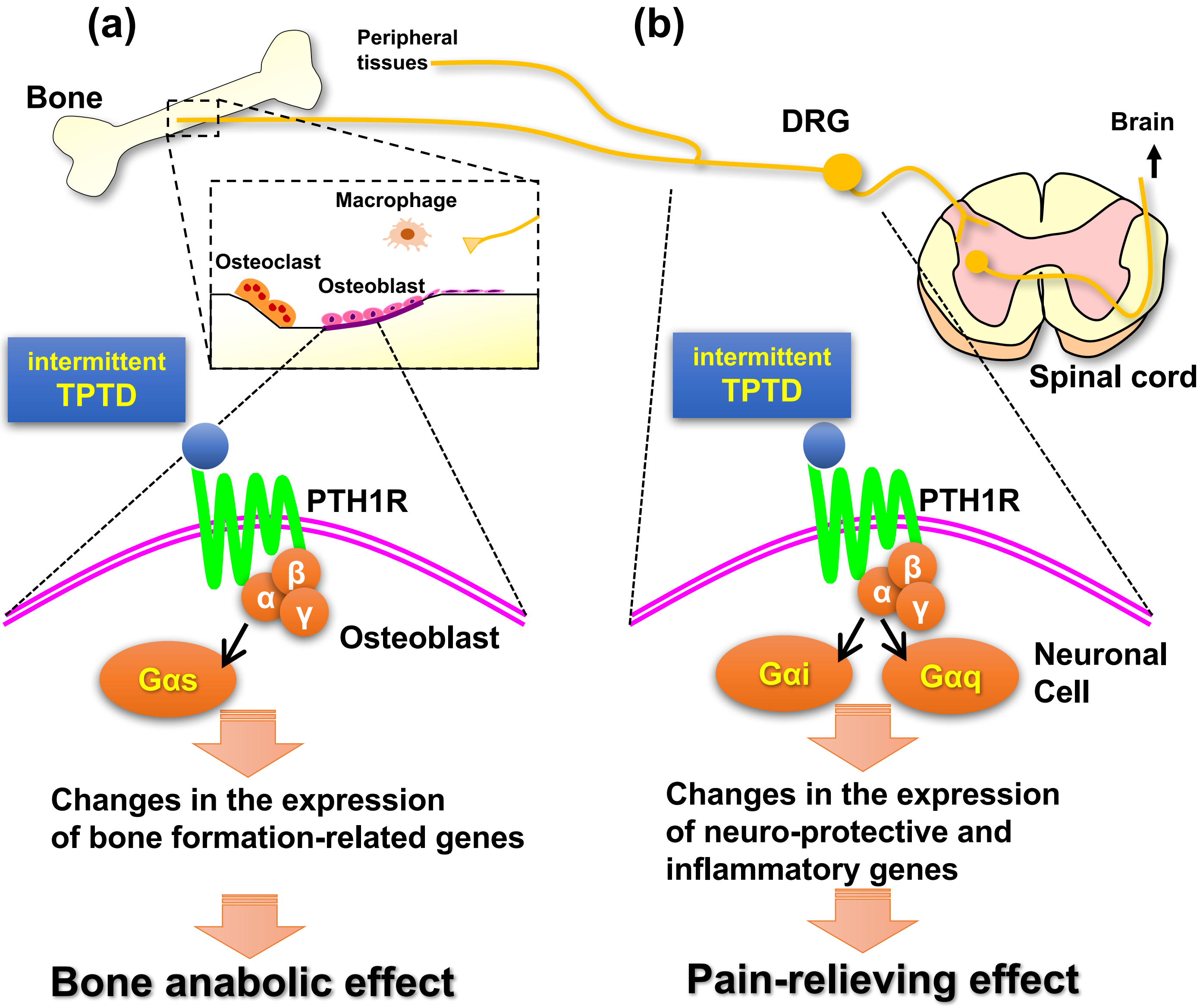

Supplementary Table.1

Gene Symbol	Gene name	Assay ID
Adra2c	adrenoceptor alpha 2C	Rn00593341_s1
Bdnf	brain-derived neurotrophic factor	Rn02531967_s1
Calca	calcitonin-related polypeptide alpha	Rn01511353_g1
Calcrl	calcitonin receptor-like	Rn00562334_m1
Ccl21	chemokine (C-C motif) ligand 21	Rn01764651_g1
Gal	galanin/GMAP prepropeptide	Rn00583681_m1
Gapdh	glyceraldehyde-3-phosphate dehydrogenase	Rn01775763_g1
Grm8	glutamate receptor, metabotropic 8	Rn00573505_m1
Il11	interleukin 11	Rn00591721_m1
Il2rg	interleukin 2 receptor, gamma	Rn01752908_g1
Kcnj5	potassium channel, inwardly rectifying subfamily J, member 5	Rn01789221_mH
Ntf3	neurotrophin 3	Rn00579280_m1
P2rx1	purinergic receptor P2X, ligand-gated ion channel, 1	Rn00564454_m1
P2ry14	purinergic receptor P2Y, G-protein coupled, 14	Rn02532502_s1
Penk	proenkephalin	Rn00567566_m1
Ptger2	prostaglandin E receptor 2 (subtype EP2)	Rn00579419_m1
Pth1r	parathyroid hormone 1 receptor	Rn00571595_m1
Stoml3	stomatin (Epb7.2)-like 3	Rn01494610_g1
Tac1	tachykinin, precursor 1	Rn01500392_m1

Supplementary Figure Legends

Supplementary Figure 1. The effect of TPTD on spinal microglia. (a) Large images of whole tissue sections of spinal cord are shown in differential interference contrast (DIC) and anti-Iba-1 immunostaining (shown in red) counterstained with DAPI (shown in blue). White boxes indicate Regions of interest (ROI) of 400 μm x 400 μm for fluorescence morphometry analyses (Figure 4). (b) Specific staining by anti-Iba-1 antibodies was confirmed by comparison with negative control staining with non-specific IgGs.

Supplementary Figure 2. The validation of the expression of *Pth1r* in DRG by PCR. Values are relative to bone samples and presented as the means ± S.E.M.

Supplementary Figure 3. Detection of PTH2R in DRG neurons. (a) PTH2R was detected by fluorescence immunohistochemistry in DRGs, including NF200-positive (a marker of myelinated Aδ fiber neurons) and NF200-negative neurons. (b) Higher magnification of the rectangle-bordered area shown in (a). (c) Klüver-Barrera staining of the same section as shown in (b) for a morphological comparison.

Supplementary Figure 4. Detection of PTH1R in bone tissue. (a) PTH1R was detected by fluorescence immunohistochemistry in osteoblastic cells covering bone surface (indicated by arrows). (b) PTH2R was not detected in bone tissue.

Supplementary Figure 5. Detection of PTH1R and CGRP in DRG neurons. Specific staining by antibodies against PTH1R and CGRP were confirmed by comparison with negative control staining with non-specific IgGs. Images are shown in DIC and immunofluorescence against PTH1R (in green) and CGRP (in red) counterstained with DAPI (in blue).

Supplementary Figure 6. A heatmap of enriched terms across input gene lists, colored by p-values. Metascape was used for the gene set enrichment analysis. (a) The DEGs between the Sham-vehicle group and the OVX-vehicle group were associated with tissue remodeling (GO:0048771) and neuroactive ligand-receptor interaction (KEGG:hsa04080). (b) The DEGs between the OVX-vehicle group and the OVX-TPTD group were associated with cytokine-mediated signaling pathways (GO:0019221) and the regulation of the response to cytokine stimuli (GO:0060759). (c) Sixteen genes were identified with statistical significance and fold change both between Sham-vehicle and OVX-vehicle and between OVX-vehicle and OVX-TPTD (see also Figure 6). The gene expression profiles are presented as a heatmap.

Supplementary Figure 7. Functional categorization of 17 genes that are the DEGs associated with the pain and inflammation. To functionally annotate and cluster these 17 genes, we conducted the Metascape enrichment analysis as described above. The gene functional annotation obtained by this analysis is shown in a hierarchical clustering heatmap (a) and two-dimensional functional mapping by t-SNE (b). Black arrows indicate the changes in gene expression by OVX. Red arrows indicate the changes in gene expression by TPTD

Supplementary Figure 8. Possible mechanism underlying the pharmacological effects of TPTD on bone cells (a) and primary sensory neurons (b).

Supplementary Table 1. The TaqMan Gene Expression assays used in this study.
